# Dysregulation of Krüppel-like Factor 2 and Myocyte Enhancer Factor 2D Drive Cardiac Microvascular Inflammation and Dysfunction in Diabetes

**DOI:** 10.3390/ijms24032482

**Published:** 2023-01-27

**Authors:** Mostafa Samak, Andreas Kues, Diana Kaltenborn, Lina Klösener, Matthias Mietsch, Giulia Germena, Rabea Hinkel

**Affiliations:** 1 Laboratory Animal Science Unit, Leibniz-Institut für Primatenforschung, Deutsches Primatenzentrum GmbH, Kellnerweg 4, 37077 Göttingen, Germany; msamak@dpz.eu (M.S.); akues@dpz.eu (A.K.); dkaltenborn@dpz.eu (D.K.); lkloesener@dpz.eu (L.K.); mmietsch@dpz.eu (M.M.); ggermena@dpz.eu (G.G.); 2 DZHK (German Centre for Cardiovascular Research), Partner Site Göttingen, 37075 Göttingen, Germany; 3 Institute for Animal Hygiene, Animal Welfare and Farm Animal Behaviour, University of Veterinary Medicine, 30173 Hannover, Germany

**Keywords:** diabetes, endothelial function, inflammation, Krüppel-like factors, myocyte enhancer factor, miR-92a, large animal models

## Abstract

Cardiovascular complications are the main cause of morbidity and mortality from diabetes. Herein, vascular inflammation is a major pathological manifestation. We previously characterized the cardiac microvascular inflammatory phenotype in diabetic patients and highlighted micro-RNA 92a (miR-92a) as a driver of endothelial dysfunction. In this article, we further dissect the molecular underlying of these findings by addressing anti-inflammatory Krüppel-like factors 2 and 4 (KLF2 and KLF4). We show that KLF2 dysregulation in diabetes correlates with greater monocyte adhesion as well as migratory defects in cardiac microvascular endothelial cells. We also describe, for the first time, a role for myocyte enhancer factor 2D (MEF2D) in cardiac microvascular dysfunction in diabetes. We show that both KLFs 2 and 4, as well as MEF2D, are dysregulated in human and porcine models of diabetes. Furthermore, we prove a direct interaction between miR-92a and all three targets. Altogether, our data strongly qualify miR-92a as a potential therapeutic target for diabetes-associated cardiovascular disease.

## 1. Introduction

Diabetes impacts the vasculature and predisposes patients to cardiovascular disease [1]. Inflammation is a hallmark of the diabetic endothelium [2]. The cardiac microvasculature is one organ system, among others, that is impacted by the diabetic inflammatory milieu and displays a well-characterized phenotype of endothelial dysfunction [3]. In this regard, we recently reported a prominent inflammatory phenotype in diabetic human cardiac microvascular endothelial cells (HCMECs) [4]. A plethora of inflammatory mediators and signaling pathways have been reported to underlie the conspicuous state of endothelial dysfunction in diabetes [5]. Most recently, we reported dysregulated gene expression of the anti-inflammatory Krüppel-like factors (KLFs) KLF2 and KLF4 in diabetic HCMECs [4]. KLFs are classified under the zinc finger family of transcription factors; 17 identified members of this subfamily have wide tissue distribution and functions [6]. KLF2 and KLF4, along with KLF6, are two of the three KLF members that are highly enriched in the endothelium [7,8]. Both KLF2 and KLF4 have been thoroughly studied in the context of inflammation as transcriptional regulators of multiple antioxidant, anti-inflammatory, and antithrombotic downstream targets [9]. Indeed, several lines of evidence from clinical and experimental studies have attributed vascular protective effects to KLF2 and KLF4 [10,11]. Expression of KLF2 was shown to be ablated in atheroprone regions of human carotid arteries [12]. Herein, KLF2 expression in the endothelial lining was dependent on flow dynamics, where laminar shear stress induced and stabilized KLF2 expression [12,13]. Tuning of KLF2 expression has been shown to be dysregulated upon oscillatory atherogenic flow [14]. Similarly, disturbed hemodynamic flow has been shown to thwart KLF4 expression both in vitro and in vivo [15]. One way in which the regulation of *KLF2* and *KLF4* gene expression operates in response to hemodynamic flow is through a family of upstream transcription factors called myocyte enhancer factor 2 (MEF2) [16]. MEF2 constitute a family of transcription factors that are crucial for development, morphogenesis, and homeostasis of the cardiovascular system [17]. Four members of the MEF2 family of transcription factors, MEF2A, -B, -C, and -D, have been identified in humans, with homologues in vertebrates [18,19]. Of particular interest to adult cardiovascular homeostasis are MEF2A and MEF2AD, which have been shown to be the predominant isoforms in the adult heart and to exhibit partial endothelial redundancy [20,21]. Studies have shown a critical role of the MEF2 family in the induction of *KLF2* and *KLF4* downstream of laminar flow, both in vitro and in vivo [21,22,23]. Moreover, endothelial-specific deletions of MEF2 lead to severe vascular phenotypes, similar to those induced by endothelial-specific deletions of *Klf2* and *Klf4*, including systemic inflammation and endothelial dysfunction [21]. However, whether MEF2 dysregulation is involved in diabetic microvascular dysfunction is not yet understood. 

In line with hemodynamic impacts on KLF gene expression, studies have attributed a role of vasoactive micro-RNA 92a (miR-92a) directly targeting *KLF2* and *KLF4* downstream of pathological hemodynamic stress, albeit in human umbilical vein endothelial cells (HUVECs) [14]. MiR-92a is a member of a polycistronic family of micro-RNAs, i.e., miR-17~92a, that have important implications in cardiovascular physiology and pathology [24]. MiR-92a has been shown to have antiangiogenic roles in several models; its inhibition confers protection and promotes recovery from ischemic injury [25,26,27]. With regard to diabetes, we previously reported the role played by miR-92a in both in vitro and in vivo diabetic models [3,4]. We showed that primary diabetic HCMECs overexpress miR-92a and that inhibition of miR-92a ameliorated the inflammatory phenotype [4]. However, whether direct interaction between miR-92a and KLF2 and/or KLF4 plays a role in the pathogenesis of the inflammatory phenotype in the diabetic cardiac microvasculature has yet to be elucidated. 

In this study, we attempt to answer the aforementioned open questions by addressing the triad of miR-92a, KLFs, and MEF2 in the context of diabetic microvascular inflammation and dysfunction using human and mouse endothelial cell models and linking it to the in vivo situation in a large animal model.

## 2. Results

### 2.1. Expression of KLF2 and KLF4 in Diabetic HCMECs and Porcine Heart Tissue

**Figure 1:**
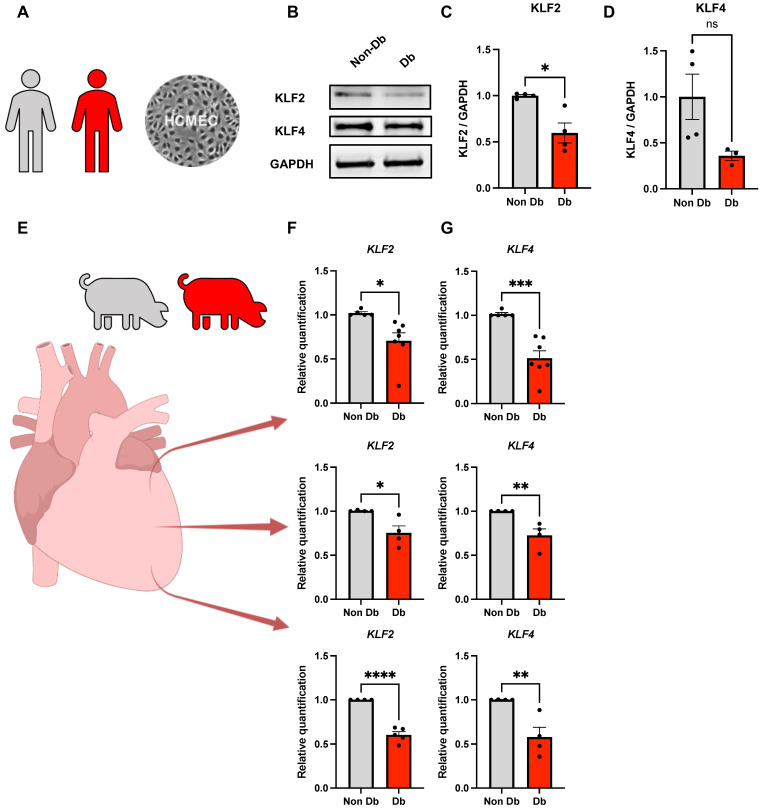
Expression of KLF2 and KLF4 in diabetic HCMECs and diabetic pig hearts. (**A**) Primary HCMECs isolated from nondiabetic or type 2 diabetic males. (**B**–**D**) Western blot analysis of relative expression and representative blot of KLF2 and KLF4 in protein lysates from nondiabetic or diabetic donors normalized to GAPDH as a housekeeping protein (n = 4). (**E**) Samples from ventricular tissue of nondiabetic or *INS*^C94Y^ diabetic pigs over the cardiac long axis. (**F**,**G**) Quantitative PCR analysis of *KLF2* and *KLF4* from base to apex, respectively. Statistical analyses by unpaired Student’s *t*-test (*n* ≥ 3); * *p* < 0.05; ** *p* < 0.01; *** *p* < 0.001; **** *p* < 0.0001.

We previously showed that diabetic HCMECs display significant reductions in *KLF2* and *KLF4* gene expression [4]. In this study, we support our data by analyzing KLF2 and KLF4 protein expression via Western blot in human primary diabetic HCMECs (Figure 1A). Indeed, KLF2 was significantly ablated in diabetic HCMEC lysates compared to nondiabetic controls (Figure 1B,C). However, KLF4 protein expression levels were not significantly different in diabetic HCMECs compared to nondiabetic controls, despite showing some trend (Figure 1B,D).

Accordingly, analysis of *KLF2* and *KLF4* gene expression in left ventricular tissue samples in a diabetic *INS*^C94Y^ transgenic porcine model showed a significant reduction in both genes over the long ventricular axis from base to apex (Figure 1E–G).

### 2.2. Endothelial Inflammatory Phenotype Driven by KLF2 Ablation

**Figure 2:**
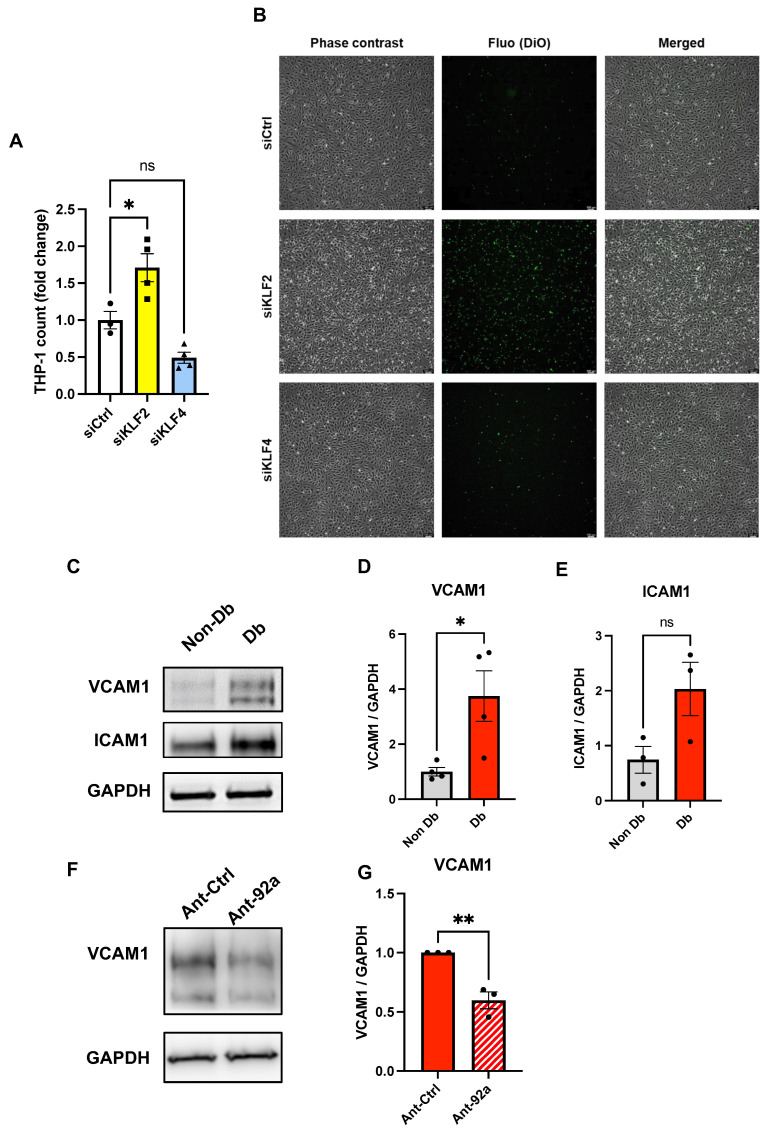
Endothelial inflammatory phenotype driven by *KLF2* ablation. (**A**) Analysis of fold change in adherent THP-1 count in nondiabetic HCMEC monolayers upon knockdown of *KLF2* (siKLF2) or *KLF4* (siKLF4) relative to controls (siCtrl) (*n* = 4). (**B**) Representative phase contrast, fluorescence, and merged images of HCMEC monolayers and DiO-labeled THP-1 (green). Scale bars equal 100 µm. (**C**) Western blot and analysis of relative protein expression of VCAM1 (**D**) and ICAM1 (**E**) in diabetic HCMECs relative to nondiabetic controls (GAPDH is considered a housekeeping protein) (*n* = 4). (**F**,**G**) Western blot analysis of relative expression and representative blot of VCAM1 in protein lysates from diabetic HCMECs upon miR-92a inhibition by antagomir (Ant-92a) relative to controls (Ant-Ctrl) normalized to GAPDH as a housekeeping protein. Statistical analyses by unpaired Student’s *t*-test or by one-way ANOVA (*n* ≥ 3); * *p* < 0.05; ** *p* < 0.01.

We previously showed that diabetic HCMECs display a pronounced inflammatory phenotype [28]. To verify whether KLF2 and/or KLF4 have anti-inflammatory properties in CMECs, we knocked down both genes in nondiabetic HCMECs (Supplementary Figure S1) and subjected the cells to flow chamber assays with unstimulated THP-1 monocytes. Herein, *KLF2* ablation resulted in a significant increase in adherent THP-1 monocytes on HCMEC monolayers compared to the controls (Figure 2A,B). Interestingly, no significant differences were observed in adherent THP-1 count upon endothelial *KLF4* knockdown (Figure 2A,B). 

Previous studies have highlighted the role of KLFs in regulating downstream inflammatory molecules in ECs [29]. These include vascular cell adhesion molecule 1 (VCAM1) and intercellular adhesion molecule 1 (ICAM1) [29]. To determine whether upregulation of these inflammatory adhesion proteins can explain the observed phenotype in diabetic HCMECs, we analyzed VCAM1 and ICAM1 relative protein levels in lysates from nondiabetic or diabetic HCMECs (Figure 2C). Indeed, VCAM1 was significantly upregulated in diabetic HCMEC protein lysates (Figure 2D), whereas the trend in ICAM1 relative increase did not meet statistical significance (Figure 2E). To evaluate the potential effect of miR-92a downregulation on the expression of the aforementioned adhesion molecules, we treated diabetic HCMECs with an antagomir against miR-92a (Ant-92a). As expected, miR-92a downregulation decreased VCAM1 expression compared to the treated controls, indicating that miR-92a has a role in the modulation of adhesion molecules.

### 2.3. The Role of miR-92a in Expression and Regulation of KLF2 and KLF4 in Endothelial Cells

**Figure 3:**
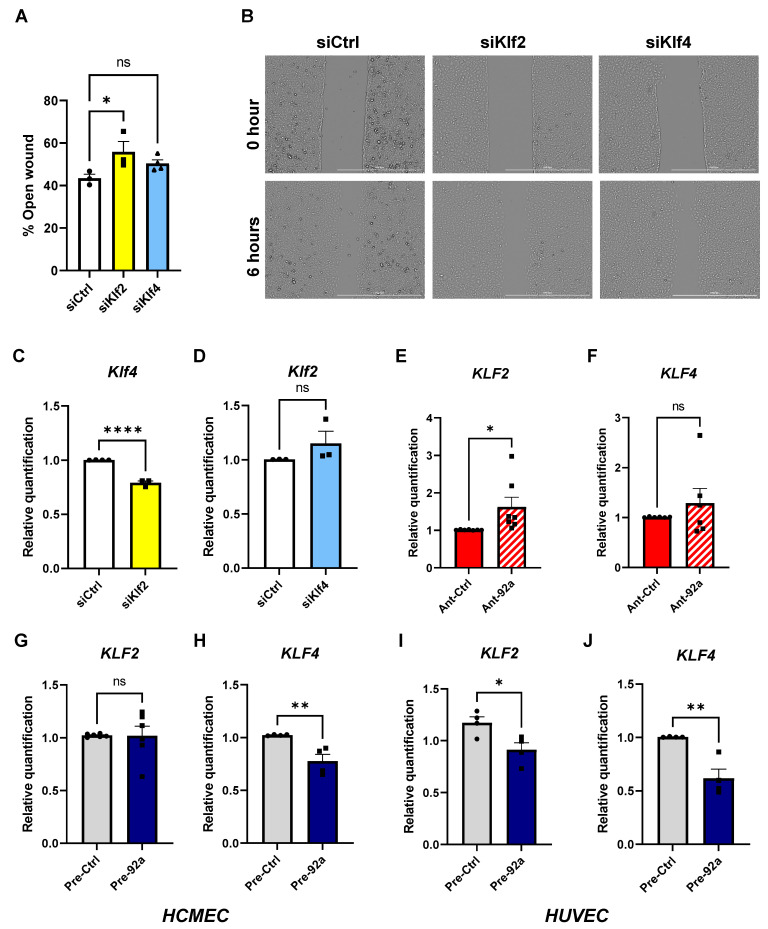
Effect and inter-regulation of *KLF2* and *KLF4* on CMEC function. (**A**) Analysis of 6 h open wound areas in percentage in MCMECs upon knockdown of *Klf2* or *Klf4* (*n* = 4). (**B**) Bright field images using a Cytation 1 plate reader of MCMEC migration at 0 and 6 h upon knockdown of *Klf2*. Scale bars equal 1 mm. Quantitative PCR analysis of (**C**) *Klf4* gene expression upon knockdown of *Klf2* in MCMECs and (**D**) *Klf2* gene expression upon knockdown of *Klf4* in MCMECs, each compared to respective controls (*n* = 4). (**E**,**F**) Quantitative PCR analysis of (**E**) *KLF2* and (**F**) *KLF4* in diabetic HCMECs upon miR-92a inhibition by antagomir (Ant-92a) relative to controls (Ant-Ctrl) (*n* = 6) and (**G**) *KLF2* and (**H**) *KLF4* in nondiabetic HCMECs upon miR-92a overexpression (pre-92a) relative to controls (pre-Ctrl) (*n* = 4). (**I**,J) Quantitative PCR analysis of (**I**) *KLF2* and (**J**) *KLF4* in HUVECs upon miR-92a overexpression (pre-92a) relative to controls (pre-Ctrl). Statistical analyses by unpaired Student’s *t*-test or by one-way ANOVA (*n* ≥ 4); * *p* < 0.05; ** *p* < 0.01; **** *p* < 0.0001; ns: not significant.

To investigate the role of KLFs in endothelial migration, we performed wound healing assays with MCMECs upon knockdown of *Klf2* or *Klf4*. Again, only the knockdown of *Klf2* correlated with a significant decrease in MCMEC migration, as evident from larger wound areas at 6 h of migration compared to controls (Figure 3A,B). However, there were no statistically significant differences in open wound areas at 6 h of migration in *Klf4*-ablated MCMECs compared to controls (Figure 3A,B).

Previous studies have alluded to the redundancy between *Klf2* and *Klf4* [30,31]; we attempted to test this redundancy in our model. Indeed, while knockdown of *Klf2* correlated with a significant reduction in *Klf4* mRNA levels, knockdown of *Klf4* did not lead to significant changes in *Klf2* expression levels (Figure 3C,D). 

To investigate whether modulation of miR-92a displays an effect on the expression levels of these KLFs under diabetic conditions, we analyzed the expression levels of both genes in diabetic HCMECs upon miR-92a antagonism (Supplementary Figure S2A). MiR-92a ablation correlated with a significant increase in *KLF2* expression levels in diabetic HCMECs relative to controls (Ant-Ctrl) (Figure 3E). However, the *KLF4* gene expression level was not significantly altered upon miR-92a inhibition in diabetic HCMECs (Figure 3F). Interestingly, miR-92a overexpression by pre-miR transfection (pre-92a) in nondiabetic HCMECs (Supplementary Figure S2B) did not impact *KLF2* expression levels (Figure 3G), whereas *KLF4* was significantly reduced relative to controls (pre-Ctrl) (Figure 3H). On the other hand, in HUVECs, miR-92a overexpression significantly impacted the expression levels of both genes (Figure 3I,J). 

### 2.4. Direct Targeting of KLF2, KLF4, and MEF2D by miR-92a

**Figure 4:**
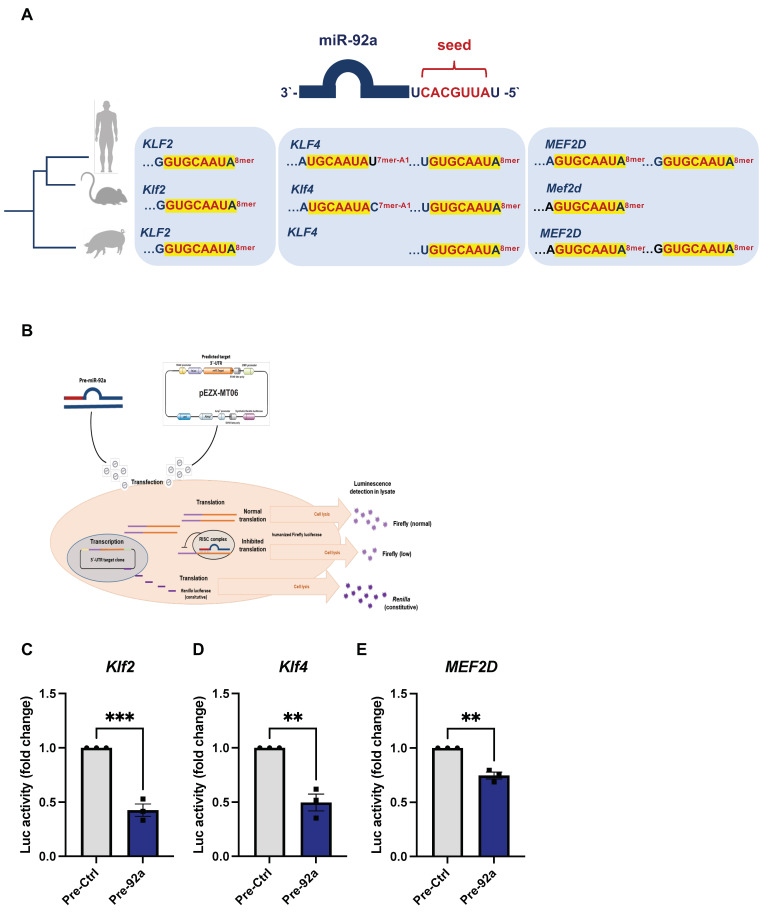
Evolutionary conservation and direct targeting of *KLF2*, *KLF4*, and *MEF2D* 3`-UTRs by miR-92a. (**A**) In silico analysis results of evolutionarily conserved target sequence of miR-92a seed sequence in *KLF2*, *KLF4*, and *MEF2 D* in humans, mice, and pigs highlighting binding sites and complementarity. (**B**) Depiction of dual luciferase reporter assay of direct interaction between miR-92a and its targets. (**C**–**E**) Dual luciferase reporter assay of (**C**) *Klf2*, (**D**) *Klf4*, and (**E**) *MEF2D* 3`-UTR reporters, respectively analyzed for fold-change in luciferase activity upon miR-92a overexpression (pre-92a) relative to controls (pre-Ctrl). Statistical analyses by unpaired Student’s *t*-test (*n* = 3); ** *p* < 0.01; *** *p* < 0.001.

*In silico* analysis of miR-92a-predicted targets by TargetScan showed conserved complementary target sequences in *KLF2*, *KLF4*, and *MEF2D* in both humans and mice (Figure 4A). Interestingly, human *MEF2D* showed two target sequences, whereas mouse *Mef2d* 3′-UTR bears one target sequence to the miR-92a seed sequence (Figure 4A). Table 1 lists the site types and context scores calculated for each target in both human (*Homo sapiens*) and mouse (*Mus musculus*), and the site types in pig (*Sus scrofa*). TargetScan provides exhaustive comparisons of micro-RNA target sites of conserved targets in different species; however, context score calculations are provided for a selected number of species. We conducted a search for conserved miR-92a target sequences in porcine *KLF2*, *KLF4*, and predicted *MEF2D* mRNAs in TargetScan and the Nucleotide Database of the National Library of Medicine. The compiled search results are depicted in Figure 4A. Indeed, evolutionarily conserved miR-92a target sequences were found in the three porcine orthologues. Unlike humans and mice, porcine *KLF4* mRNA bears one miR-92a target site with 8mer complementarity and lacks the 7mer-A1 target site present in both humans and mice. Like humans, however, predicted porcine *MEF2D* showed two miR-92a target sites in its 3`-UTR (Figure 4A).

To test the direct interaction between miR-92a and the aforementioned predicted targets, we conducted dual luciferase reporter assays with mammalian expression vectors of the respective 3′-UTR of mouse *Klf2* or *Klf4* or human *MEF2D* downstream of Firefly luciferase and Renilla luciferase as endogenous control (Figure 4B). Upon cotransfection of pre-miR-92a, there were significant reductions in reporter activity of all three targets, as evident from the calculated Firefly to Renilla luciferase activity (FLuc/RLuc) relative to control transfected cells (pre-Ctrl) (Figure 4C–E).

### 2.5. MEF2D as miR-92a Target Dysregulated in Diabetes

**Figure 5:**
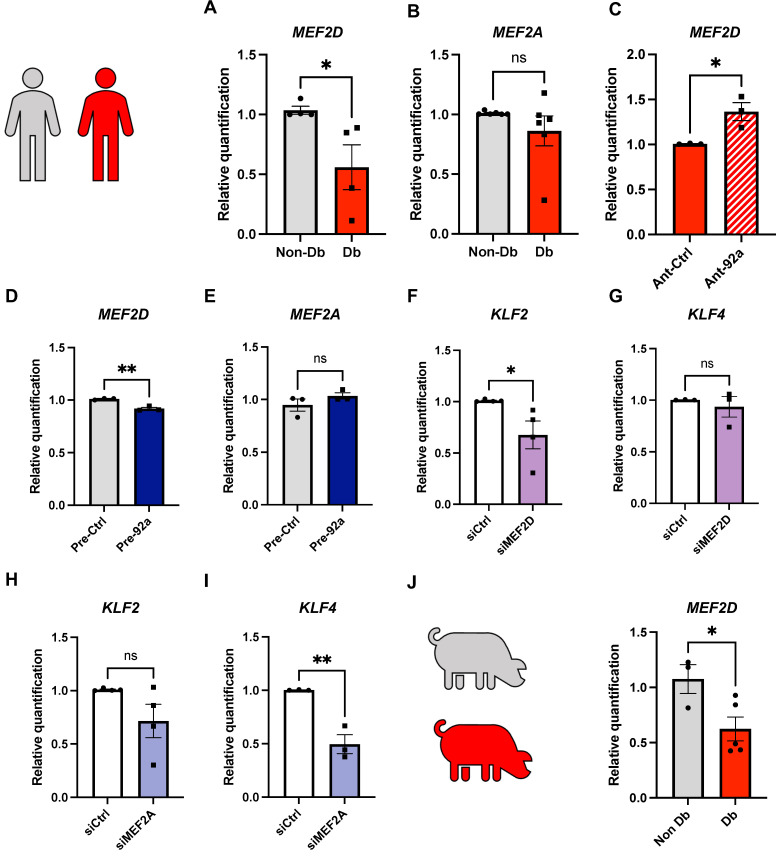
Regulation of *MEF2D* in diabetes and by miR-92a. (**A**,**B**) Quantitative PCR analysis of relative expression of *MEF2D* and *MEF2A* in nondiabetic vs. diabetic HCMECs (*n* = 4), and of (**C**) *MEF2D* in diabetic HCMECs upon miR-92a antagonism (Ant-92a) relative to its respective control (Ant-Ctrl) (*n* = 3). (**D**,**E**) Quantitative PCR analysis of *MEF2D* and *MEF2A* expression in nondiabetic HCMECs upon miR-92a overexpression (pre-92a) relative to controls (pre-Ctrl) (*n* = 3). (**F**–**I**) Quantitative PCR analysis of (**F**) *KLF2* and (**G**) *KLF4* gene expression upon knockdown of *MEF2D* and (**H**) *KLF2* and (**I**) *KLF4* gene expression upon knockdown of *MEF2A*, each compared to respective controls (*n* = 5). (**J**) Quantitative PCR analysis of *MEF2D* expression in diabetic *INS^C94Y^* pigs relative to their nondiabetic littermates (*n* = 5). Statistical analyses by unpaired Student’s *t*-test; * *p* < 0.05; ** *p* < 0.01.

As both MEF2A and MEF2D are crucial for vascular homeostasis, we investigated their gene expression in our diabetic models. As expected, *MEF2D* gene expression was significantly reduced in diabetic HCMECs relative to their nondiabetic counterparts (Figure 5A). *MEF2A*, however, was not significantly different (Figure 5B). To verify whether *MEF2D* levels could be restored by miR-92a inhibition, we transfected diabetic HCMECs with an antagomir to miR-92a and analyzed *MEF2D* gene expression by quantitative PCR. Like *KLF2*, miR-92a ablation led to a significant increase in *MEF2D* mRNA level relative to controls (Ant-Ctrl) (Figure 5C). Moreover, overexpression of miR-92a in nondiabetic HCMECs resulted in a slight, yet significant, reduction in the *MEF2D* expression level relative to controls (Figure 5D). *MEF2A* expression levels, on the other hand, were not altered upon mir-92a changes (Figure 5E).

MEF2 transcription factors have been reported to be the master regulators of KLF2 and KLF4 expression in endothelial cells downstream of hemodynamic flow. To determine whether this applies to our CMECs and whether the observed dysregulation of KLF expression in diabetes is linked to the observed *MEF2D* dysregulation in our diabetic models, we knocked down *MEF2A* or *MEF2D* in nondiabetic HCMECs (Supplementary Figure S3A,B) and analyzed *KLF2* and *KLF4* gene expression. Indeed, mimicking diabetic conditions, knockdown of *MEF2D* led to significant downregulation in *KLF2*, but not in *KLF4*, gene expression (Figure 5F,G). Interestingly, knockdown of *MEF2A* led to significant downregulation of *KLF4* but not of *KLF2* (Figure 5H,I). Neither did knockdown of *MEF2D* or *MEF2A* affect the expression levels of each other (Supplementary Figure S4A,B).

Consistent with these findings, *MEF2D* expression was also significantly reduced in diabetic porcine ventricular tissue (Figure 5J).

## 3. Discussion

Diabetes-induced vascular dysfunction is an indisputable clinical finding and is the main culprit in cardiovascular-related morbidity and mortality in diabetic patients [1,32]. A compilation of data from clinical and translational research has emphasized the role of inflammation in the pathogenesis of diabetic vascular dysfunction [2,33]. Herein, the cardiac microvasculature is especially vulnerable in diabetes [3,34,35]. In our previous study, we characterized the inflammatory phenotype of diabetic cardiac microvascular endothelial cells [4]. We reported dysregulation in two prominent anti-inflammatory transcription factors, KLF2 and KLF4, in diabetic HCMECs using gene expression analysis. These two Krüppel-like factors are known to be enriched in the endothelium and regulate key genes therein, maintaining vascular integrity and homeostasis. Here, we showed that KLF2 is downregulated at the protein level in diabetic HCMECs and on gene expression levels throughout the left ventricular tissue of diabetic porcine models. The congruence between two different diabetic models in the two species highlights the importance of KLF2 as a common denominator in the diabetic cardiovascular phenotype. Indeed, previous work by Hinkel et al. that characterized myocardial tissue alterations in diabetic pig models clearly reflects the situation in human diabetic hearts [3]. Although KLF4 was dysregulated at the gene expression level in both diabetic HCMECs and porcine ventricular tissue, this was not the case at the protein level in HCMECs—despite some trend. Given the tight inter-regulation and partial redundancy between both KLFs and the reported catastrophic outcomes of losing them both in the endothelium, maintenance of some activity of one in the absence of the other is essential and can be viewed as an adaptation [30,36,37].

The functional consequences of dysregulated KLF2 were tested in this study, and the results indicate that dysregulated KLF2 can well explain the diabetic inflammatory phenotype we previously reported, i.e., increased monocyte adhesion to endothelial monolayers. Moreover, endothelial migration was also negatively affected. Again, *KLF4* knockdown alone was not sufficient to elicit the same response. This clearly highlights the greater importance of KLF2 in this regard and well explains the reported evolutionary protection of this crucial factor. A study by Sweet et al. [28] addressed this bioinformatically, with strong evidence showing a remarkable increase in the GC content of *KLF2* in later evolved species, culminating with strikingly high values in mammalian *KLF2* [31]. Moreover, their results show a dramatic increase in KLF2 binding motifs in primates, with humans at the top of this trend. This highlights the great importance, and hence the grave outcomes, of KLF2 dysregulation. Restoring KLF2 function in vascular disease conditions, such as diabetes, is therefore expected to be of therapeutic value. 

One way KLF2 has an anti-inflammatory function is by thwarting NF-κB downstream expression of adhesion molecules; it does so by transcriptionally activating transcription factor EB (TFEB), which interferes with the inflammatory NF-κB cascade [29]. Here, we showed that VCAM1 levels are indeed significantly upregulated in diabetic HCMECs and upon KLF2 ablation, whereas ICAM1 is not significantly increased. The latter finding is in congruence with other reports that KLF2 does not significantly impact ICAM1 [38]. 

We previously reported the role of miR-92a in the pathogenesis of cardiac microvascular dysfunction in diabetes [28]. Both *KLF2* and *KLF4* are predicted miR-92a targets. Interestingly, unlike in HUVECs, miR-92a overexpression in nondiabetic HCMECs did not influence *KLF2* gene expression, which can be understood in light of the aforementioned tight regulation of *KLF2*. Furthermore, this highlights the endothelial heterogeneity between HUVECs and HCMECs, which should advise further research endeavors. On the other hand, the inhibition of miR-92a in diabetic HCMECs restored *KLF2* expression levels. This can well explain our previously reported anti-inflammatory effects of miR-92a antagonism in diabetic HCMECs. It is not known, however, whether such an increase in gene expression of *KLF2* upon miR-92a antagonism also rescues protein levels. Herein, the availability of samples from primary cell lines poses limitations to our study. While miR-92a alone cannot dysregulate *KLF2* in healthy cardiac microvasculature, inhibition of micro-RNA in diabetes can bring about benefits. 

Previous reports have described the interaction between miR-92a and *KLF2* as a product of hemodynamic disturbances, where oscillatory atherogenic flow induces miR-92a and in turn downregulates *KLF2* [14]. Under normal pulsatile flow, however, *KLF2* is upregulated downstream of the MEF2 family of transcription factors [21]. Studies have shown the imperative role of MEF2 in regulating *KLF2* expression [24,39]. Interestingly, our *in silico* analysis predicted *MEF2D* as an miR-92a target with two conserved seed sequence complementary sites in both humans and pigs. As predicted, we showed using a luciferase reporter assay that miR-92a does directly target *KLF2*, *KLF4*, and *MEF2D*. Moreover, we established a link between our previously reported overexpression of miR-92a in diabetes and dysregulated *MEF2D* levels, which we have shown here in both diabetic HCMECs and diabetic porcine ventricular tissue samples (Graphical Summary). Furthermore, the MEF2 family of transcription factors has been shown to be crucial for angiogenesis by regulating the expression of the Notch family of genes [40]. Their dysregulation in diabetic HCMECs and diabetic porcine ventricles can further explain our previously described cardiac microvascular angiogenic defects in diabetes [4]. Of note, miR-92a inhibition also restored *MEF2D* levels in diabetic HCMECs, thereby explaining the previously reported proangiogenic role of miR-92a inhibitors in diabetic HCMECs [4].

One limitation of our study is the limited number of HCMEC donors from nondiabetic or diabetic backgrounds. This is an issue with primary cell culture that should be addressed with further large-scale experimental design to include more patients. A second limitation is the lack of in vivo data concerning the effectiveness of miR-92a inhibitors in diabetic models of coronary microvascular dysfunction. 

From a clinical perspective, miRNA inhibitors have recently been gaining greater interest as novel therapeutic molecules for cardiovascular disease; many have undergone and completed phase II clinical trials [41]. Herein, a miR-92a inhibitor, MRG-110, has recently been tested for clinical safety and efficacy in healthy volunteers, with promising results [42].

## 4. Materials and Methods

### 4.1. EC Culture

Primary ventricular HCMEC (PromoCell, Heidelberg, Germany C-12286), 51-year-old male (Lot #470Z011.7), 63-year-old nondiabetic (Lot no. 447Z026.3), or 63-year-old type 2 diabetic (Lot no. 451Z015.1) Caucasian males were cultured in PromoCell microvascular media MV (C-22020) or MV2 (C-22022), supplemented with their corresponding supplement mixes (C-39225 or C-39226, respectively) and 0.1% penicillin/streptomycin (PS). Cells were kept in a humidified incubator at 37 °C and 5% CO₂ and used for experiments between passages 2 and 8.

Mouse cardiac microvascular endothelial cells (MCMEC) (Cedarlane, Burlington, ON, Canada CLU510) were cultured according to the provider’s instructions in DMEM with 10 mM, 10 mmol/L HEPES, 1% PS, and 5% fetal bovine serum (FBS).

THP-1 monocytes (ATCC, Manassas, VA, United States TIB-202) were maintained in RPMI medium with 10% FBS, 0.05 mM 2-mercaptoethanol, and 1% PS.

### 4.2. Transfection

Cells in the culture were transfected at 80% confluence in MV2 (HCMEC) or serum-free DMEM (MCMEC) with Lipofectamine RNAiMAX reagent (Invitrogen, Waltham, Massachusetts, United States —13778150) and small RNA at a concentration of 10 nM using the manufacturer’s protocol. Transfection complexes were prepared in OptiMEM™ (Gibco, Dreieich, Germany) medium, added, and incubated with the cells for 4–6 h, followed by medium change and incubation for an additional 24 h in complex-free medium before the cells were used for angiogenesis assays. For all other assays, the cells were collected by trypsinization directly after the following small RNA (Ambion, Invitrogen, Waltham, Massachusetts, United States) were used: Anti-miR™ hsa-miR-92a-3p (Ant-92a) (AM10916); anti-miR™ Negative Control (Ant-Ctrl) (AM17010); pre-miR™ miRNA precursors hsa-miR-92a (PM10916) and mmu-miR-92a (PM10312) (Pre-92a); pre-miR™ miRNA precursor negative controls (Pre-Ctrl) (AM17110/AM17111); Silencer^®^ siRNA against *KLF2* (4392420, ID: s20269); *KLF4* (4392420, ID: s17793); *Klf2* (4390771, ID: s68830); *Klf4* (4390771, ID: s68837); *MEF2D* (4392420, ID: s8656); *MEF2A* (4427037, ID: s230700); or negative controls (4390844/4390846).

### 4.3. Wound Healing

The wound healing assay was performed using 2-well culture inserts for self-insertion (Ibidi, Gräfelfing, Germany - 80209). The inserts were placed in 6-well plates (TPP^®^, Trasadingen, Switzerland - 92006), and 70 μL of cell suspensions of 5 × 10^5^ cells/mL were added to each well of the 2-well inserts. The cells were incubated for 36 h. The inserts were then carefully removed, the cells were washed with calcium- and magnesium-free phosphate buffer saline (PBS), and fresh medium was added. Time-lapse imaging of wound healing was performed using a BioTek Cytation 1^TM^ multimode reader (Agilent, Santa Clara, California, United States), where several bright field 5 × microscopic images were taken at time intervals over 6 h. Automatic analysis of the wound areas was performed using the machine’s software. 

### 4.4. Flow Chamber Assay

HCMEC suspensions were prepared at 5 × 10^5^ cells/mL, and 40 μL were added to each channel of a μ-Slide VI0.4 (Ibidi, Gräfelfing, Germany - 80606). The cells were incubated for 24–36 h to form stable monolayers. The flow chamber experiment was adapted from Stachel et al., 2013 [43]. Briefly, THP-1 monocytes were stained with Vybrant^TM^ Cell-Labeling Solutions DiI (V-22885) or DiO (V-22886) (Thermo Fisher Scientific, Waltham, Massachusetts, United States) according to the manufacturer’s protocol. The cells were washed with PBS, and a concentration of 7.5 × 10^5^ cells/mL was prepared in their culture medium. A perfusion system was established; two 50 mL Original-Perfusor^®^ syringes (B. Braun, Melsungen, Germany) were filled with THP-1 monocyte cell suspension or MV2 medium (washing medium), connected with perfusion lines to a 3-way valve, and each placed in Perfusor^®^ Space pumps (B. Braun, Melsungen, Germany). A tube adapter set (Ibidi, Gräfelfing, Germany 10831) was connected to the valve and in the flow chamber inlets. A flow of 47.4 mL/hour was established to simulate venous shear stress 1 dyn/cm^2^, and a flow round was run for each channel as follows: 2-min washing medium, 5-min cell suspension, and 2-min washing medium. The flow slide was then imaged using phase contrast and fluorescent imaging. 

### 4.5. Western Blot

The cells were collected by trypsinization, centrifuged at 250 RCF for 5 min, washed with calcium- and magnesium-free PBS, and centrifuged at 10,000 rpm for 5 min. The cells were lysed for 10 min on ice in lysis buffer (10 mM Tris/HCl pH 7.5, 150 mM NaCl, 0.5 mM EDTA, and 0.5% NP-40) containing a protease and phosphatase inhibitor cocktail (Roche, Basel, Switzerland). After clarification, the protein concentration was quantified, and the different samples were resuspended in sample buffer. Western blots were performed, and immunoblots were incubated with the following antibodies to: GAPDH (Cell Signaling, Danvers, MA, USA - 97166); KLF2 (Thermo Fisher Scientific, Waltham, MA, USA - PA5-40591); KLF4 (Cell Signaling 4038); VCAM1 (Santa Cruz Dallas, TX, USA - sc-13160); or ICAM1 (Santa Cruz sc-8439) as primary antibodies, and Horseradish-Peroxidase-linked secondary anti-mouse (Cell Signaling 7076S) or anti-rabbit (Cell Signaling 7074S) antibodies. Signals were developed by treatment with enzymatic chemiluminescence (ECL) reagents (Amersham™, Amersham, United Kingdom - RPN 2232), imaged using a ChemiDoc™ Imaging System from Biorad (Hercules, CA, USA), and analyzed using Image Lab 6.1 software from Biorad. Adjusted band volumes for target proteins were normalized to those of GAPDH as a housekeeping control.

### 4.6. Quantitative PCR

The cells were collected by trypsinization, centrifuged at 2000 rpm for 5 min, and small and/or large RNA was extracted using a NucleoSpin^®^ miRNA kit (Macherey & Nagel, Düren, Germany). For gene expression analysis, 500 ng RNA was used for cDNA synthesis by Omniscript^®^ Reverse Transcription kit (Qiagen, Hilden, Germany 205113). Quantitative PCR was run using TaqMan™ Fast Advanced Master Mix (Applied Biosystems, Waltham, Massachusetts, United States - 4444557) and the following TaqMan assays (primers): human beta-actin (*ACTB*) (Hs99999903_m1); *KLF2* (Hs00360439_g1); *KLF4* (Hs00358836_m1); *Klf2* (Mm00500486_g1); *Klf4* (Mm00516104_m1); *MEF2A* (Hs01050409_m1); *MEF2D* (Hs00954735_m1); mouse beta-actin (Actb) (Mm02619580_g1); *Klf2* (Mm00500486_g1); and *Klf4* (Mm00516104_m1). MiR-92 quantification was performed as described earlier [28]. A quantity of 20 ng of RNA was used for cDNA synthesis using a TaqMan™ Advanced miRNA cDNA Synthesis Kit (A28007) and TaqMan™ Advanced miRNA Assay (A25576) and the following assays for human hsa-miR-92a-3p (assay ID 477827_mir) and hsa-miR-26a-5p (assay ID 477995_mir) as endogenous controls. Real-time PCR (RT-PCR) was run using the recommended thermal cycling profiles and StepOnePlus™ software v2.3 (Applied Biosystems, Waltham, Massachusetts, United States) to calculate the comparative CT (relative quantification).

Myocardial tissue from *INS^C94Y^* transgenic diabetic pigs and nondiabetic littermates was obtained from the Institute of Molecular Animal Breeding and Biotechnology, Gene Center, LMU Munich [3]. Those pigs harbor an insulin mutation causing a Cys → Tyr exchange at position 94 that resembles the human mutation, INS^C96Y^, which is associated with permanent neonatal diabetes mellitus (PNDM) [44,45]. The mutation disrupts a sulfide bond between the A and B chains of the insulin molecule, leading to a misfolded insulin structure and impaired secretion. They exhibit early postnatal hyperglycemia and significantly depleted pancreatic β-cell mass due to apoptosis at 4.5 months of age, representing a model of type 1 diabetes without the autoimmune component [45]. Porcine *INS^C94Y^* myocardial tissue was dissociated in ML buffer from NucleoSpin^®^ miRNA kit (Macherey & Nagel, Düren, Germany), gentleMACS™ Dissociator, and M-tubes (Miltenyi Biotec, Bergisch Gladbach, Germany). RNA extraction and gene expression analysis were performed as previously described using the following TaqMan assays (primers): pig beta-actin (*ACTB*) (Ss03376563_uH); *KLF2* (Ss06942161_g1); *KLF4* (Ss03391985_m1); and *MEF2D* (Ss06884968_m1).

### 4.7. ImageJ Analysis

Adherent THP-1 to endothelial monolayers in flow chambers was counted by particle number quantification in ImageJ. Briefly, images were split into three color components, and depending on the dye (DiO), the red- or green-colored image was subjected to threshold adjustment. Particle analysis settings were applied, and the particles were counted.

### 4.8. Statistical Analysis

Data were analyzed using GraphPad Prism or Microsoft Excel 2016 software and presented as mean ± SEM (error bars). Sample size and experimental replicates were indicated in figure legends. Statistical analyses were performed using Student’s *t*-test (two groups) or one-way ANOVA (three or four groups). *p*-values, * *p* < 0.05; ** *p* < 0.01; *** *p* < 0.001; and **** *p* < 0.0001 were considered statistically significant, whereas “ns” denotes not statistically significant differences.

## 5. Conclusions

This study follows up on our previously published findings on cardiac microvascular inflammation in diabetes and highlights the molecular interactions in diabetic coronary microvascular dysfunction. Both KLF2 and KLF4 have previously been reported in vascular inflammation and as targets of miR-92a. In this study, we provide evidence for the involvement of KLF2 in microvascular inflammation in diabetic hearts. Moreover, to the best of our knowledge, this is the first report on the possible contribution of *MEF2D* dysregulation to the pathogenesis of diabetic cardiac microvascular dysfunction upstream of KLF2. However, more experiments are needed to further characterize MEF2 in diabetes. Importantly, derepression of these crucial vascular factors by miR-92a antagomir provides a molecular elaboration of our previously reported proangiogenic and anti-inflammatory effects of miR-92a inhibitors in diabetic cardiac microcirculation, as well as in other models of cardiovascular injury reported by others, e.g., ischemia/reperfusion injury and atherosclerosis [26,46,47]. Evidence from both porcine models of type 1 diabetes and primary HCMECs from type 2 diabetes enhances our understanding of the ubiquitous aspects of these signaling pathways in cardiac microvasculature both in vivo and in vitro. While some research questions are currently under investigation by our group, the presented data provide strong support for the clinical utility of miR-92a inhibitors as modulators of cardiovascular disease in diabetes.

## Figures and Tables

**Table 1:** In silico analysis results of miR-92a predicted downstream targets by TargetScan.

Target mRNA	Match Position	Site Type	Site Context Score
*Homo sapiens*-*KLF2*	242–249	8mer	−0.50
*Homo sapiens*-*KLF4*	362–368	7mer-A1	−0.06
	674–681	8mer	−0.41
*Homo sapiens*-*MEF2D*	858–865	8mer	−0.28
	2814–2821	8mer	−0.09
*Mus musculus*-*Klf2*	214–221	8mer	−0.57
*Mus musculus*-*Klf4*	433–439	7mer-A1	−0.10
	751–758	8mer	−0.36
*Mus musculus*-*Mef2d*	1057–1064	8mer	−0.24
*Sus scrofa*-*KLF2*	NA	8mer	NA
*Sus scrofa*-*KLF4*	NA	8mer	NA
*Sus scrofa*-*MEF2D*	NA	8mer	NA
		8mer	

NA: not available in TargetScan.

## Data Availability

Not applicable.

## Supplementary Materials

The following supporting information can be downloaded at: https://www.mdpi.com/1422-0067/24/3/2482/s1.
