# Correction: Samak et al. Dysregulation of Krüppel-like Factor 2 and Myocyte Enhancer Factor 2D Drive Cardiac Microvascular Inflammation and Dysfunction in Diabetes. *Int. J. Mol. Sci.* 2023, *24*, 2482

**DOI:** 10.3390/ijms25021058

**Published:** 2024-01-15

**Authors:** Mostafa Samak, Andreas Kues, Diana Kaltenborn, Lina Klösener, Matthias Mietsch, Giulia Germena, Rabea Hinkel

**Affiliations:** 1 Laboratory Animal Science Unit, Leibniz-Institut für Primatenforschung, Deutsches Primatenzentrum GmbH, Kellnerweg 4, 37077 Göttingen, Germany; 2 DZHK (German Centre for Cardiovascular Research), Partner Site Göttingen, 37075 Göttingen, Germany; 3 Institute for Animal Hygiene, Animal Welfare and Farm Animal Behaviour, University of Veterinary Medicine, 30173 Hannover, Germany

In the original publication [1], there was a mistake in Figure 3 as published. The bar graphs in A, C, E, F, H, I, and J were missing the asterisks denoting the statistical significance of the pairwise comparison in correspondence to the *p* values in the figure legend. The corrected Figure 3 appears below. The authors state that the scientific conclusions are unaffected. This correction was approved by the Academic Editor. The original publication has also been updated.

## Figures and Tables

**Figure 3:**
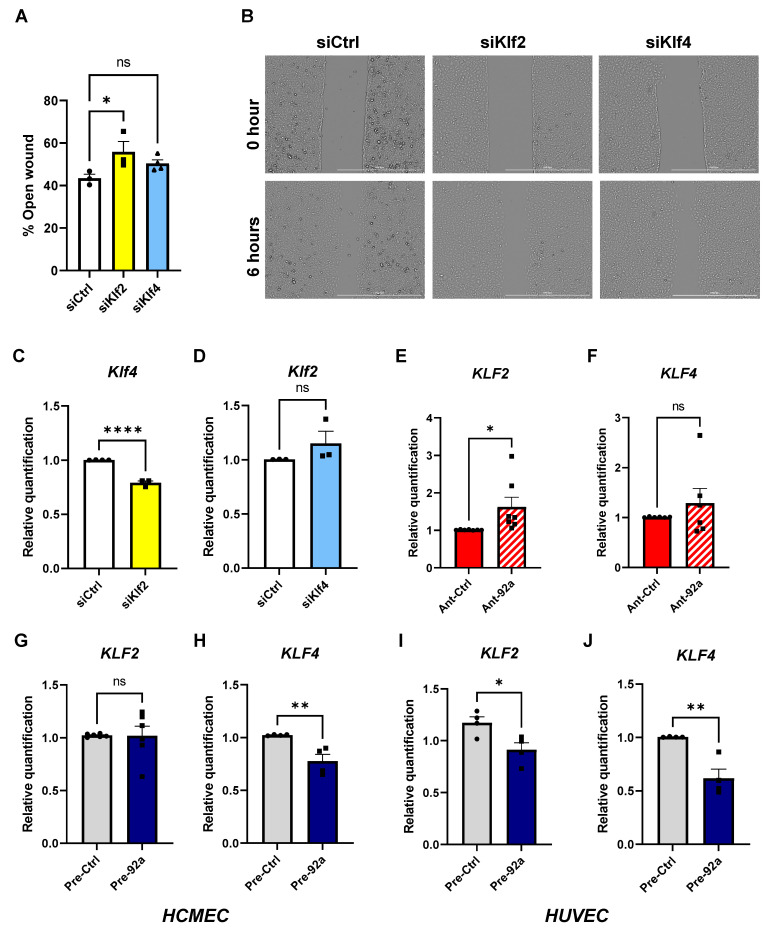
Effect and inter-regulation of *KLF2* and *KLF4* on CMEC function. (**A**) Analysis of 6 h open wound areas in percentage in MCMECs upon knockdown of *Klf2* or *Klf4* (*n* = 4). (**B**) Bright field images using a Cytation 1 plate reader of MCMEC migration at 0 and 6 h upon knockdown of *Klf2*. Scale bars equal 1 mm. Quantitative PCR analysis of (**C**) *Klf4* gene expression upon knockdown of *Klf2* in MCMECs and (**D**) *Klf2* gene expression upon knockdown of *Klf4* in MCMECs, each compared to respective controls (*n* = 4). (**E**,**F**) Quantitative PCR analysis of (**E**) *KLF2* and (**F**) *KLF4* in diabetic HCMECs upon miR-92a inhibition by antagomir (Ant-92a) relative to controls (Ant-Ctrl) (*n* = 6) and (**G**) *KLF2* and (**H**) *KLF4* in nondiabetic HCMECs upon miR-92a overexpression (pre-92a) relative to controls (pre-Ctrl) (*n* = 4). (**I**,**J**) Quantitative PCR analysis of (**I**) *KLF2* and (**J**) *KLF4* in HUVECs upon miR-92a overexpression (pre-92a) relative to controls (pre-Ctrl). Statistical analyses by unpaired Student’s *t*-test or by one-way ANOVA (*n* ≥ 4); * *p* < 0.05; ** *p* < 0.01; **** *p* < 0.0001; ns: not significant.
