# Supplementary material for: Dysregulation of Krüppel-like Factor 2 and Myocyte Enhancer Factor 2D Drive Cardiac Microvascular Inflammation and Dysfunction in Diabetes
Source: Int J Mol Sci. 2023 Jan 27;24(3):2482. doi: 10.3390/ijms24032482 (PMC9916909; doi:10.3390/ijms24032482)
Supplement: Supplementary file 1 [file ijms-24-02482-s001.zip › ijms-2143446-supplementary.pdf]

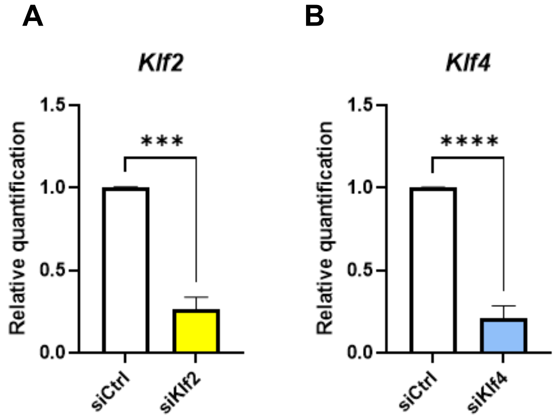

Figure S1: Evidence of knockdown of *Klf2* and *Klf4* in MCMEC by specific siRNA. (A, B) Quantitative PCR analysis of expression of *Klf2* and *Klf4* in MCMEC upon transfection of corresponding specific siRNA (siKlf2, siKlf4), respectively, relative to controls (siCtrl). Statistical analyses by unpaired student t-test (n = 4); *<0.05; **<0.01; ***<0.001; ****<0.0001.

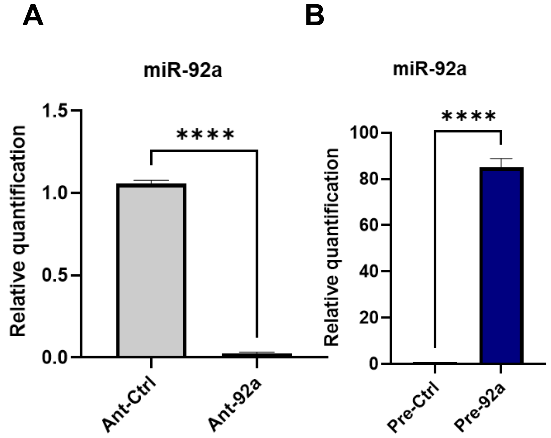

figure S2: Evidence of overexpression or ablation of miR-92a in HCMEC. Quantitative PCR analysis of miR-92a in HCMEC upon (B) downregulation by Ant-92a relative to control (Ant-Ctrl) or (A) overexpression by pre-92a relative to control (pre-Ctrl). Statistical analyses by unpaired student t-test (n = 5); *<0.05; **<0.01; ***<0.001; ****<0.0001

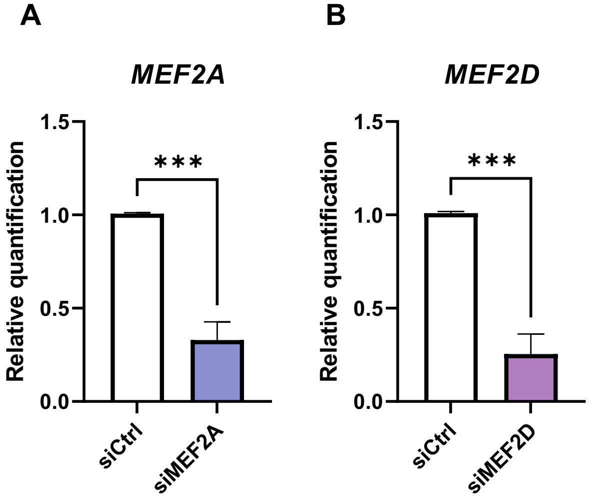

Figure S3: Evidence of knockdown of *MEF2A* or *MEF2D* in non-diabetic HCMEC. Quantitative PCR analysis of *MEF2A* or *MEF2D* in non-diabetic HCMEC upon transfection with respective siRNA (siMEF2A or siMEF2D) compared to control (siCtrl). Statistical analyses by unpaired student t-test (n = 4) ; *<0.05; **<0.01; ***<0.001; ****<0.0001.

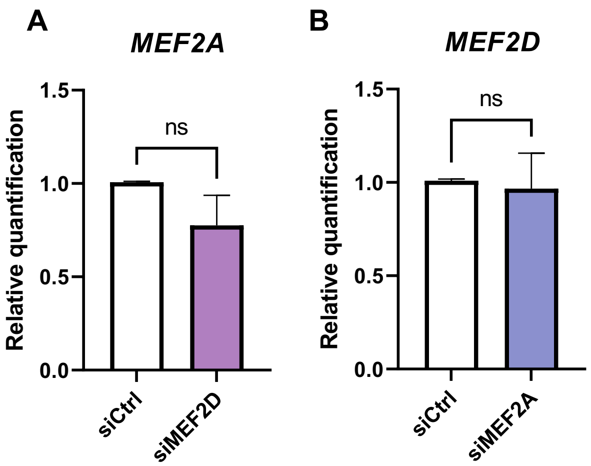

Figure S4: Quantification of *MEF2A* or *MEF2D* in non-diabetic HCMEC upon *MEF2D* or *MEF2A* downregulation. Quantitative PCR analysis of *MEF2A* or *MEF2D* in non-diabetic HCMEC upon transfection with respective siRNA (siMEF2A or siMEF2D) compared to control (siCtrl). Statistical analyses by unpaired student t-test (n = 4); *<0.05; **<0.01; ***<0.001; ****<0.0001.
